# Concordant integrative gene set enrichment analysis of multiple large-scale two-sample expression data sets

**DOI:** 10.1186/1471-2164-15-S1-S6

**Published:** 2014-01-24

**Authors:** Yinglei Lai, Fanni Zhang, Tapan K Nayak, Reza Modarres, Norman H Lee, Timothy A McCaffrey

**Affiliations:** Department of Statistics, The George Washington University, 801 22nd St. NW. Rome Hall, Room 553, Washington, D.C 20052 USA; Department of Pharmacology, The George Washington University Medical Center, Washington, DC 20037 USA; Department of Medicine, Division of Genomic Medicine, The George Washington University Medical Center, Washington, DC 20037 USA

## Abstract

**Background:**

Gene set enrichment analysis (GSEA) is an important approach to the analysis of coordinate expression changes at a pathway level. Although many statistical and computational methods have been proposed for GSEA, the issue of a concordant integrative GSEA of multiple expression data sets has not been well addressed. Among different related data sets collected for the same or similar study purposes, it is important to identify pathways or gene sets with concordant enrichment.

**Methods:**

We categorize the underlying true states of differential expression into three representative categories: no change, positive change and negative change. Due to data noise, what we observe from experiments may not indicate the underlying truth. Although these categories are not observed in practice, they can be considered in a mixture model framework. Then, we define the mathematical concept of concordant gene set enrichment and calculate its related probability based on a three-component multivariate normal mixture model. The related false discovery rate can be calculated and used to rank different gene sets.

**Results:**

We used three published lung cancer microarray gene expression data sets to illustrate our proposed method. One analysis based on the first two data sets was conducted to compare our result with a previous published result based on a GSEA conducted separately for each individual data set. This comparison illustrates the advantage of our proposed concordant integrative gene set enrichment analysis. Then, with a relatively new and larger pathway collection, we used our method to conduct an integrative analysis of the first two data sets and also all three data sets. Both results showed that many gene sets could be identified with low false discovery rates. A consistency between both results was also observed. A further exploration based on the KEGG cancer pathway collection showed that a majority of these pathways could be identified by our proposed method.

**Conclusions:**

This study illustrates that we can improve detection power and discovery consistency through a concordant integrative analysis of multiple large-scale two-sample gene expression data sets.

## Background

The recent large-scale technologies like microarrays [1–3] and RNA-seq [4, 5] allow us to collect genome-wide expression profiles for biomedical studies. Genes showing significant differential expression are potentially important biomarkers [6]. Furthermore, a gene set enrichment analysis enables us to identify groups of genes (e.g. pathways) showing coordinate differential expression [7, 8]. For some disease studies, multiple gene expression data sets have been collected and the related integrative analysis of multiple data sets has been investigated [9]. Since microarray and sequencing based genome-wide expression data sets have been increasingly collected, it is necessary to further develop the computational and statistical methods for integrative data analysis studies.

Genes and gene sets showing consistent behavior among multiple related studies can be of great biological interest. However, since the sample sizes are usually small but the numbers of genes are large, it is difficult to identify truly differentially expressed genes and determine whether a gene or a gene set behaves concordantly among different related studies. Although the integrative analysis of multiple gene expression data sets has been well studied in recent years [10, 11], the genome-wide concordance has not been well considered. Misleading results may be generated if the concordance among different data sets is not considered in an integrative analysis. Our purpose is to identify pathways or gene sets with concordant enrichment. Recently, there are several methods published for meta gene set enrichment analysis of expression data [12, 13]. However, these methods have not been specifically developed for our study purpose. Statistically, we need analysis methods that are consistent with the study purpose. There is still a lack of methods and software for the concordant integrative gene set enrichment analysis.

For a gene set enrichment analysis, an enriched gene set in one data set may also be enriched in another data set. However, this gene set is not necessarily concordantly enriched in both data sets. For an illustration, let us consider a simple artificial example: gene set *S* contains five genes with the first three genes strongly up-regulated in the first data set (the last two genes non-differentially expressed) and the last three genes strongly up-regulated in the second data set (the first two genes non-differentially expressed). Then, in general, gene set *S* is enriched in up-regulated differential expression in both data sets. However, there is only one gene up-regulated in both data sets; the remaining genes are showing inconsistent behavior. Therefore, unless the proportions of differentially expressed genes are small, there is a lack of evidence to conclude that gene set *S* is concordantly enriched in both data sets. Since a gene set concordantly enriched in several similar studies may be of great importance, it is necessary to develop statistical methods for detecting these gene sets.

It has been shown that a mixture model based approach can be an efficient approach to the differential expression analysis [14]. Furthermore, we have also demonstrated the usefulness of mixture models in concordant analysis of differential expression among large-scale expression data sets [15, 16]. The advantage of the mixture model based approach is that the probability of a particular behavior (up-regulated or down-regulated) can be modeled and estimated for a given gene. Thus, it is feasible to address how likely this gene shows a concordant behavior. In this study, we develop a mixture model based method for a concordant integrative gene set enrichment analysis.

## Methods

### Concordant gene set enrichment

In this study, we consider multiple large-scale two-sample gene expression data sets. We use *K* to denote the number of these data sets and *m* to denote the number of common genes in these data sets. For each of these data sets, we usually use a *t*-type test to evaluate the differential expression of each gene and a gene set enrichment analysis (GSEA) method to evaluate the enrichment level of a given gene set. In order to define and evaluate a concordant gene set enrichment when an integrative analysis is conducted for all *K* data sets, we categorize differential expression in each data set into three underlying (unobserved) representative categories: no change, positive change (or up-regulated differential expression) and negative change (or down-regulated differential expression). Due to data noise, what we observe from experiments may not indicate the underlying truth. (For example, a gene with slight down-regulated differential expression may show a small positive *t*-type test value.) Although these categories are not observed in practice, they can be considered in a mixture model framework.

To understand the concept of concordant gene set enrichment, let us consider an artificial example. Given a pathway with 30 genes, we know all the underlying behavior of these genes: 20 genes have positive changes consistently among all different data sets. Furthermore, if we randomly select 30 genes, we also know that the expected number of genes with consistent positive changes among different data sets is just 5. In this case, we would conclude that the given gene set is concordantly enriched in up-regulated differential expression (because 30 is clearly larger than 5). However, in practice, all the underlying differential expression categories are not observed. Instead, they can be considered in a mixture model framework. Then, we need to develop a mathematical formula for the probability of concordant enrichment score (CES) of a given gene set *S* that contains *m*_*S*_ genes:

which can be useful for prioritizing different gene sets in practice.

Before we derive the mathematical formula for the above probability, we need to explain the term "enriched". As suggested by Efron and Tibshirani [17], unless the test statistic for a gene set enrichment analysis (GSEA) considers the genome-wide background patterns (e.g. the statistics proposed in the original GSEA [7, 8]), it is necessary to consider the "row randomization" for genes in addition to the "column permutation" for samples. Therefore, the term "enriched" means "higher/better than expected".

Although many test statistics have been developed for GSEA with one large-scale expression data set, we still need to develop a new approach for this study. The motivation is: we need to address the component information of the genes in a gene set. The component information is whether a gene is up-regulated, down-regulated or non-differentially expressed. Most existing test statistics for the gene set enrichment analysis are either nonparametric or functions of *z*-score. But it is difficult to analyze the component information with these test statistics. Therefore, based on the above discussion for the term "enriched", we propose the following probability for measuring concordant gene set enrichment:

For a gene in a given gene set *S*, an event of interest can be: (1) the gene is concordantly up-regulated; (2) the gene is concordantly down-regulated; or (3) the gene is concordantly differentially expressed (either up-regulated or down-regulated). Our analysis methods for these different types of enrichment analysis are almost mathematically identical. For a mathematical notation of the above CES, we denote *U*_*i*_ the indicator that the *i*-th gene in gene set *S* satisfies the event of interest. Let **D** be the observed data and *π* be the probability of event of interest if the gene is randomly sampled. Then, we have

In order to calculate CES practically, we propose a three-component multivariate mixture model. In the model, each component is a normal distribution. The model configuration for these three components is consistent with the differential expression categories as described above. This model is conceptually analog to a simple normal mixture approach to differential expression analysis proposed by McLachlan et al. [14]. The special feature of our model is that we focus on some specific combination of components from different dimensions. A bivariate version of this model has been used by us to evaluate the concordance and discordance between two large-scale experiments with two sample groups [15] and to integrate two microarray data sets in differential expression analysis [16]. Before the model description, we need to describe the related data preprocessing and differential expression test scores as follows.

### Data preprocessing

Because our proposed statistical method is developed based on the differential expression test scores, we assume that the given gene expression data sets have been preprocessed appropriately [18]. For a concordant integrative analysis of multiple data sets, we also need to select genes shared commonly by different data sets. This can be achieved using the genes' unique identifiers.

### Differential expression test scores

For each of the two-sample gene expression data sets, we screen individual genes with the traditional two-sample Student's *t*-test. Several modified *t*-tests, such as SAM *t*-test [19] and the moderated *t*-test [20], have been widely used in the differential expression analysis of microarray data. These test statistics can generally improve the control of false positives by "softly" filtering out genes with relatively small expression variance. However, we intend to consider all the genes equally important in the concordant integrative analysis of multiple data sets. Furthermore, a given gene can show different levels of variance in different data sets, which may make it difficult to use these modified *t*-tests. Therefore, we still recommend the traditional two-sample *t*-test as the differential expression test statistic. (In practice, other test statistics like SAM *t*-test or the moderated *t*-test can still be considered when there is a strong reason to do so.) Because the sample size of a high-throughput study is usually not large, it is generally difficult to validate the normal distribution assumptions for the *t*-test. Therefore, instead of the theoretical *t*-distribution, we use the permutation procedure to compute the *p*-value of an observed *t*-test [21]. This approach has been widely adopted in the analysis of gene expression data [6].

For *K* two-sample gene expression data sets with *m* common genes, we compute the one-sided upper-tailed *p*-value *p*_*i,k*_ for gene *X*_*i*_ in the *k*-th data set, *i* = 1, 2*, . . . , m* and *k* = 1, 2*, . . . , K*. Then, we perform an inverse normal transformation to obtain a *z*-score: *z*_*i,k*_ = Φ^*-*1^(1 *- p*_*i,k*_), where Φ(*·*) is the cumulative distribution function (c.d.f.) of the standard normal distribution. This transformation has been widely used to improve the fitting of a mixture model [14]. Our proposed statistical methods for the concordant integrative analyses of multiple data sets are developed based on these sets of *z*-scores.

### A mixture model

For each individual data set, we assume that a mixture of three normal distributions can well fit the *z*-scores. Let  denote the probability density function (p.d.f.) of a normal distribution with mean *µ* and variance *σ*^2^. Three representative components are considered for the *k*-th data set (*k* = 1, 2*, . . . , K*):  for genes non-differentially expressed (no change),  for genes with up-regulated differential expression (positive change) and  for genes with down-regulated differential expression (negative change). Notice that *µ*_0*,k*_= 0 and  (a *z*-score under the null hypothesis follows the standard normal distribution because its associated *p*-value follows a standard uniform distribution). This configuration has been suggested in the analysis of gene expression data [14] although more components can be considered to improve the data fitting. Mathematically, we have the following density function:

which is a type of well-known simple normal mixture model.

When the above simple model is extended to accommodate the analysis of multiple data sets, we need to consider the combination of components from different dimensions (data sets). Then, there are 3^*K*^ different combinations. We assume that different data sets are collected independently. For the *i*-th gene with a list of *z*-scores  from different data sets, if we know all the related component information, then the join density of these *z*-scores is the product of marginal densities of individual *z*-scores. Therefore, the following formula defines our basic mixture model for a concordant analysis:1

where  is the probability for this gene being in a particular combination of different components (*j*_1_*, j*_2_*, . . . , j*_*K*_) in different data sets . We call this model a partial concordance/discordance (PCD) model. Notice that a bivariate version of this model has been used to evaluate the overall concordance or discordance of two large-scale data sets and to conduct an integrative analysis of differential expression for two large-scale two-sample data sets [15, 16].

### Model estimation

Our mixture model can be estimated by the well-developed E-M algorithm [22]. In the model, the differential expression categories are considered as missing information. For any *z*-score vector (*z*_*i*,1_, *z*_*i*,2_, . . . , *z*_*i,K*_), *i* = 1, 2, . . . , *m*, this information can be mathematically represented as  if each *z*_*i,k*_ is sampled from the *j*_*k*_-th component (*j*_*k*_ = 0, 1 or 2 and *k* = 1, 2*, . . . , K*) or zero otherwise.

With only the observed data, the likelihood can be calculated by the following formula:

where Θ represents the parameter space described previously. The "complete likelihood" based on the observed data and missing information can be calculated by the following formula:

Then, we can derive the following E-step formula:

We can also derive the following M-step formulas:

In the E-M algorithm, we iterate E-step and M-step until a numerical convergence of likelihood (not the "complete likelihood"). Let *L*^(*t*) ^and *L*^(*t*+1) ^be the likelihood values calculated after the *t*-th and (*t* + 1)-th iterations, respectively. A numerical convergence is claimed if *|L*^(*t*+1) ^*− L*^(*t*)^*| <* 0.001.

### Concordant enrichment score

Suppose that we are interested in gene sets with coordinate up-regulated differential expression (the CES formulas for the other events of interest can be derived similarly). Then, we need to focus on the combination of different components with (*j*_1_ = 1*, j*_2_ = 1*, . . . , j*_*K*_ = 1). Based on the mixture model, we can derive the following probability for a gene *X*_*S,i*_ in a given gene set *S* = {*X*_*S,i*_ : *i* = 1, 2*, . . . , m*_*S*_}:

This probability *u*_*S,i*_ can be estimated as  by plugging-in the estimated parameters in the PCD model. Let *h*_*S,i*_ be either 0 or 1. Under the assumption that *z*-scores {*z*_*i,k*_ : *i* = 1, 2*, . . . , m*} from different genes are independent in each data set *k, k* = 1, 2*, . . . , K*, we can calculate the concordant enrichment score (CES) for a gene set *S* = {*X*_*S,i*_ : *i* = 1, 2*, . . . , m*_*S*_}:2

which is the PCD model based estimate for the probability **Pr**(gene set *S* is concordantly enriched *|* observed *z*-score matrix of gene set *S*). In the formula, *I*(true statement) = 1 and *I*(false statement) = 0 (indicator function). Notice that the formula can be simplified to a well-known binomial tail probability if all  are the same. However,  are usually different in practice. Then, we need to calculate a tail probability for a heterogeneous Bernoulli process.

For the calculation for gene sets with coordinate down-regulated differential expression, we need to focus on the combination of different components with (*j*_1_ = 2*, j*_2_ = 2*, . . . , j*_*K*_ = 2). Then, we need to change the formulas for *u*_*S,i*_ and *CES*_*S*_ as follows:

### False discovery rate

The concordant enrichment score given in Equation (2) is an estimated conditional probability of concordant enrichment, which can be considered as the true positive probability for the gene set *S*. This conditional probability is closely related to the concept of false discovery rate (FDR). FDR has been widely used to evaluate the proportion of false positives among the claimed positives [6, 23]. According to the discussion by McLachlan et al. [14], among the *J* top gene sets {*S*_1_*, S*_2_*, . . . , S*_*J*_} claimed significantly concordantly enriched, the false discovery rate can be estimated as:3

### Computational approximation

Although we have derived the formula for concordant enrichment score (CES), it is usually difficult to compute it in practice: the number of possible component combinations from different genes in a given gene set is usually huge. Based on our observation, most gene sets contain more than 20 genes. Since different genes have different probabilities of being concordantly up-regulated and/or down-regulated differentially expressed, we cannot further simplify the formula (we need to calculate a tail probability for a heterogeneous Bernoulli process). However, we can consider a simulation based approach to the approximation of CES given in Equation (2).

#### Monte Carlo approximation

Recall that the probability of event of interest *u*_*S,i*_ can be calculated for a gene *X*_*S,i*_ in a given gene set *S* = {*X*_*S,i*_*, i* = 1, 2*, . . . , m*_*S*_}. The simulation scheme is based on a heterogeneous Bernoulli process:

- For each *X*_*S,i*_, simulate a Bernoulli random variable with probability of event *u*_*S,i*_;
- For the gene set *S*, count the number *R* of events from different genes;
- Repeat the above two steps *B* times and report the approximated enrichment score as {number of .

One related question is how large *B* should be set in the simulation. As we have discussed above, the concordant enrichment score (CES) is closely related to the false discovery rate (FDR). Then, it is reasonable to require its accuracy around the 1% level for the 95% CES level (e.g. a 95% normally approximated binomial confidence interval 0.95 *±* 0.01) and *B* = 2000 is adequate. Therefore, the Monte Carlo approximation is a feasible approach in practice. (In general, if we do not have a specific CES level, we can simply use an upper bound *B* = 10000 calculated based on the 95% normally approximated binomial confidence interval. Then, the related computing burden is still practically feasible.)

## Results and discussion

### Application #1: an integrative analysis of two data sets

To illustrate our method, we first considered two microarray gene expression data sets collected for lung cancer studies [24, 25]. The first one was collected by a research group in Boston (referred to as Boston data) and the second one was collected by a research group in Michigan (referred to as Michigan data). For an application of their Gene Set Enrichment Analysis (GSEA) method, Subramanian, Tamayo et al. [8] reorganized these two data sets, which were made freely available at http://www.broadinstitute.org/gsea. There were 62 and 86 patients for the Boston and Michigan data sets, respectively. These patients were classified as either "good" or "poor" outcomes. Expression profiles were available for 5216 genes that were common for both data sets. To compare our analysis results with the results reported by Subramanian, Tamayo et al. [8], we used an early version of gene set collection that was used by them [8]. Subramanian, Tamayo et al. [8] also suggested a moderate range of 15-500 genes for the sizes of gene sets that were analyzed in their gene set analysis. A gene set was not analyzed if its number of genes was out of this range. This range was used in our analysis. To demonstrate the advantage of their GSEA, Subramanian, Tamayo et al. [8] observed several commonly significantly enriched gene sets from the analysis of each data set although no individual genes with significantly differential expression were identified.

Since no concordant integrative analysis has been conducted before for these two data sets, it is necessary to investigate whether more significant results can be achieved by such an analysis. Lai et al. [15] and Lai et al. [16] have discussed that it is necessary to evaluate the genome-wide concordance before an integrative analysis to be conducted. Based on a likelihood ratio test [15, 16], we obtained *p*-values *<* 0.01 and *>* 0.3 for testing hypothesis complete discordance (CD) model vs. partial concordance/discordance (PCD) model and complete concordance (CC) model vs. PCD model, respectively. This result suggested that the expression profiles of both data sets were overall concordant at a genome-wide level. To avoid any possible selection bias, we still conducted our integrative analysis based on the general PCD model. (When the simplified CC model was used, we still observed similar results [not shown].) As shown in Table 1, the gene sets identified by Subramanian, Tamayo et al. [8] were also identified by our method. Furthermore, the resulting false discovery rates (FDRs) were even more significant (all below 0.08 and most of them below 0.001) by our method. [The complete gene sets (328 gene sets) with the false discovery rates (FDRs) based on our concordant integrative gene set enrichment analysis have been included in our supplementary material.]

**Table 1:** A comparison based on two data sets.

Gene sets enriched in poor outcome	FDR
Boston data	
Hypoxia and p53 in the cardiovascular system	*<*0.001
Aminoacyl tRNA biosynthesis	*<*0.001
Insulin upregulated genes	*<*0.001
tRNA synthetases	*<*0.001
Leucine deprivation down-regulated genes	*<*0.001
Telomerase up-regulated genes	*<*0.001
Glutamine deprivation down-regulated genes	*<*0.001
Cell cycle checkpoint	*<*0.001
Michigan data	
Glycolysis gluconeogenesis	*<*0.001
vegf pathway	*<*0.001
Insulin up-regulated genes	*<*0.001
Insulin signaling	0.021
Telomerase up-regulated genes	*<*0.001
Glutamate metabolism	0.018
Ceramide pathway	0.076
p53 signalling	*<*0.001
tRNA synthetases	*<*0.001
Breast cancer estrogen signalling	*<*0.001
Aminoacyl tRNA biosynthesis	*<*0.001

Gene sets identified by Subramanian, Tamayo et al. [8] for Boston and Michigan data are listed with the false discovery rates (FDRs) calculated by our proposed concordant integrative gene set enrichment analysis. Based on the gene set enrichment analysis (GSEA) for each individual data set, the FDRs calculated by Subramanian, Tamayo et al. [8] were between 0.006 to 0.25 (most of them were between 0.1 to 0.2).

**Figure 1:**
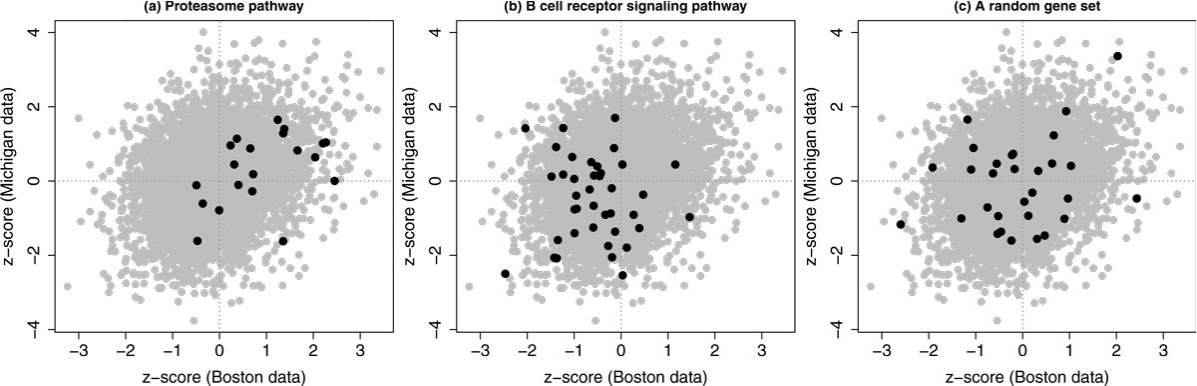
**Illustrative examples based on two data sets**. Three illustrative examples for our proposed method for a concordant integrative gene set enrichment analysis of two data sets. In each plot, the gray dots represent all paired z-scores for 5216 common human genes and the black dots represent the paired z-scores for the gene set specified in the title.

Figure 1 gives some graphical illustrative examples for our concordant integrative gene set enrichment analysis. Proteasome degradation is a well-known pathway in cancer studies [26]. Furthermore, proteasome inhibitors are being used clinically in lung cancer treatments [27]. Yang et al. [28] has also demonstrated that proteasome regulates the key survival factors for cells. However, this gene set had not been identified in the study by Subramanian, Tamayo et al. [8]. As shown in Figure 1, the majority of *z*-scores from both data sets were positive for the gene set proteasome pathway. Because most of these *z*-scores were relatively close to zero, it was difficult to identify this pathway by an analysis based on individual data sets. However, the concordant enrichment of up-regulation of this gene set was identified by our integrative analysis approach (CES > 0.999 and FDR < 0.001 for the up-regulation enrichment). The B cell receptor (BCR) signaling pathway has been shown to be important in immune disease and cancer studies [29]. As shown in Figure 1, the majority of *z*-scores from both data sets were negative for this gene set although these *z*-scores were also relatively close to zero. For the down-regulation enrichment, the CES and FDR for this gene set was *>* 0.9 and ~ 0.05, respectively. For a comparison, we also randomly selected 30 genes as a random gene set. As shown in Figure 1, the paired *z*-score pattern of this random gene set was consistent with the genome-wide paired *z*-score distribution. Therefore, this random gene set was not significantly concordantly enriched. (The corresponding up-regulation and down-regulation based CES were both in the range of 0.4 ~ 0.6.)

**Figure 2:**
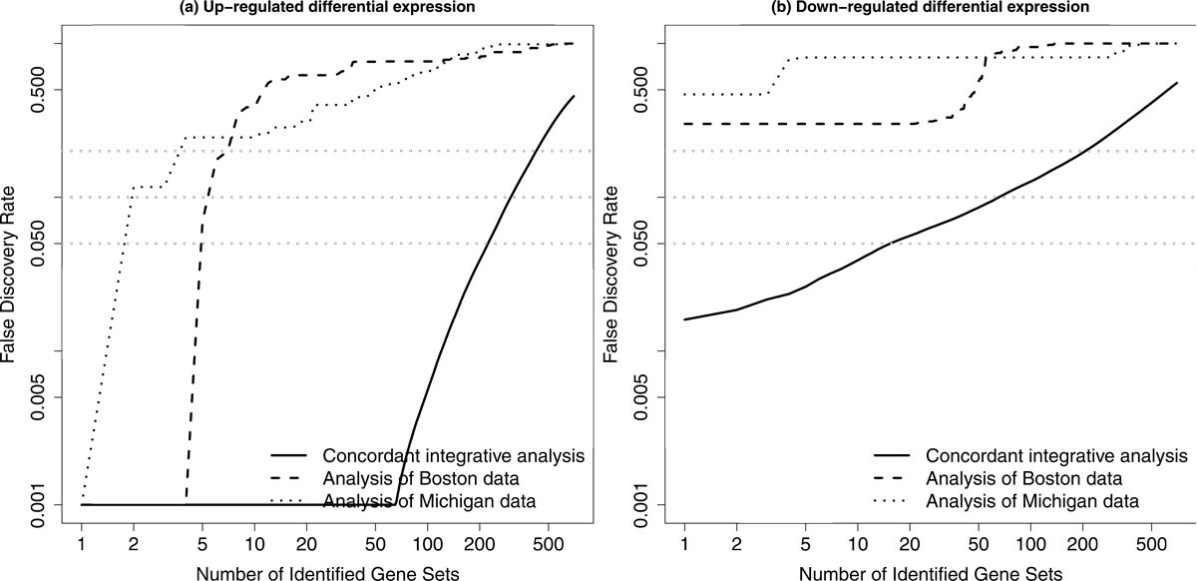
**A comparison of FDR curves based on two data sets**. A comparison of false discovery rate (FDR) curve based on our proposed method for concordant integrative gene set enrichment analysis with the FDR curves based on the gene set analysis (GSA) for individual data sets. In each plot, the black solid curve represents the results based on our method; the black dashed curve represents the results based on GSA for the Boston data; the black dotted curve represents the results based on GSA for the Michigan data. The gray dotted lines represent three FDR levels: 0.05, 0.1 and 0.2. Both up-regulated (a) and down-regulated (b) differential expression based analysis results are presented.

Mootha, Lindgren, et al. [7] have made their gene set collections freely available at their web site for Molecular Signatures Database. Since the introduction of gene set enrichment analysis [7, 8], the collections of gene sets have been updated to version 3.0 (at the time of our study). Therefore, based on the Boston and Michigan data, we have performed a concordant integrative analysis for this updated version of the C2 canonical pathway collection. Among 880 gene sets in the collection, there are 700 gene sets with gene number range from 15 to 500. We have also compared our results with the results calculated separately for individual data sets based on the gene set analysis (GSA) method proposed by Efron and Tibshirani [17]. Although certain statistical assumptions are required for the GSA method, Maciejewski [30] have still suggested in a recent comparison study that this method is one of the preferred methods for a gene set enrichment analysis. Figure 2 shows that the false discovery rate (FDR) curve based on our method is clearly lower than these two FDR curves based on GSA (one for Boston data and the other for Michigan data). There are 224 and 15 pathways with significant CES (FDR < 0.05) for up-regulated and down-regulated differential expression, respectively. These results have been included in our supplementary material.

### Application #2: an integrative analysis of three data sets

**Figure 3:**
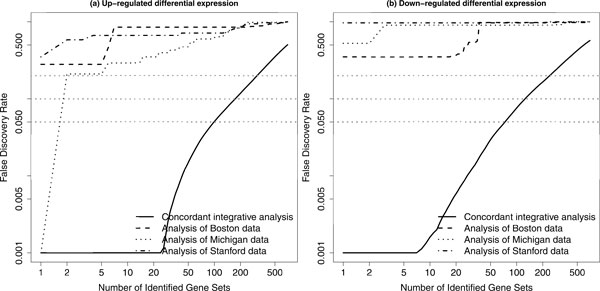
**A comparison of FDR curves based on three data sets**. A comparison of false discovery rate (FDR) curve based on our proposed method for concordant integrative gene set enrichment analysis with the FDR curves based on the gene set analysis (GSA) for individual data sets. In each plot, the black solid curve represents the results based on our method; the black dashed curve represents the results based on GSA for the Boston data; the black dotted curve represents the results based on GSA for the Michigan data; the black dot-dashed curve represents the results based on GSA for the Stanford data. The gray dotted lines represent three FDR levels: 0.05, 0.1 and 0.2. Both up-regulated (a) and down-regulated (b) differential expression based analysis results are presented.

In addition to the Boston and Michigan data sets, Subramanian, Tamayo et al. [8] also mentioned and reorganized another related data set collected by a Stanford study [31]. We then considered these three data sets together for a concordant integrative gene set enrichment analysis. The number of patients in the Stanford data set was much less: 24 patients were classified as either "good" or "poor" outcomes. For these three data sets, there were 2865 common genes (almost 50% reduction from the first application described above). We still used the Version 3.0 of the C2 canonical pathway collection. The GSA method was again used to analyze individual data sets separately for 700 gene sets (see above for details). Figure 3 shows that the FDR curve based on our method is still clearly lower than these three FDR curves based on GSA (one curve for each data set). There are 99 and 74 pathways with significant CES (FDR < 0.05) for up-regulated and down-regulated differential expression, respectively. These results have also been included in our supplementary material.

Among the gene sets with FDR < 0.05, we observed many interesting pathways. Among these 74 identified based on down-regulated differential expression, there were pathways related to immune system, TCR signaling, viral myocarditis, BCR signaling, cell survival, WNT-*β*-catenin signaling, cytokine, PI3K, VEGF signaling, interleukins and GPCR signaling. Among these 99 identified based on up-regulated differential expression, there were pathways related to different metabolism, cell cycle, checkpoints, and related phases and transitions, DNA replication, synthesis damage and repair, p53, glycolysis gluconeogenesis, telomere maintenance and extension, apoptosis, TGF-*β* signaling, tRNA aminoacylation, gene expression, lung cancer and PDGF signaling.

### Consistency between two application results

**Figure 4:**
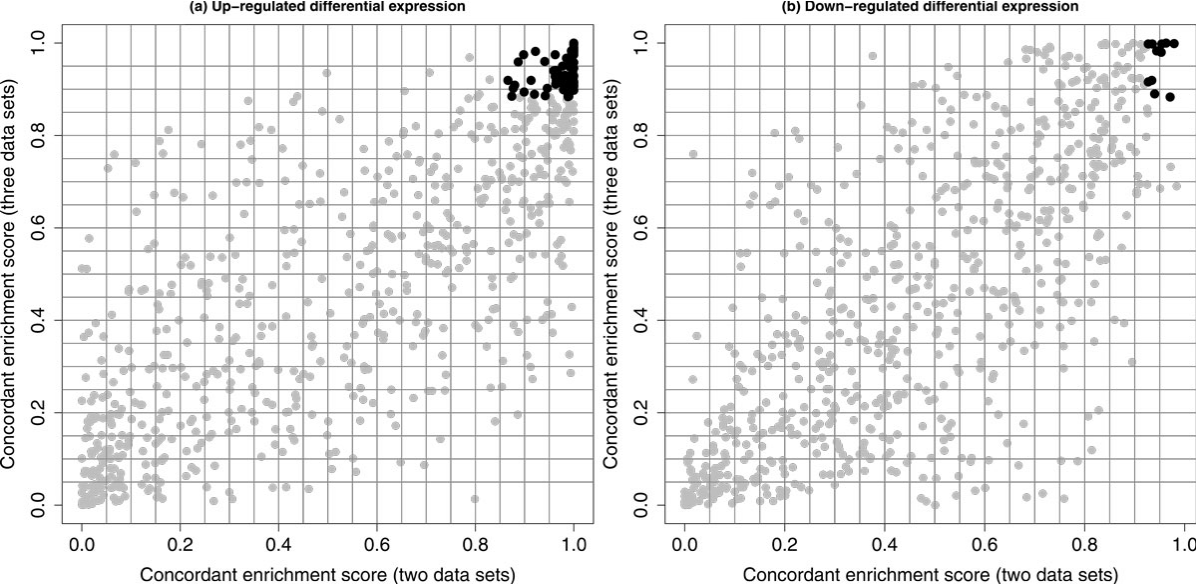
**A comparison of CESs based on two application results**. A comparison of our concordant integrative gene set enrichment analysis results based on two data sets to the results based on three data sets. In each plot, the gray dots represent the paired concordant enrichment scores (CESs) for all pathways in the Version 3.0 of the C2 canonical pathway collection and the black dots represent the paired CESs for pathways with FDR< 0.05 for both analysis results. Both up-regulated (a) and down-regulated (b) differential expression based analysis results are presented.

We also investigated whether the inclusion of an additional data set to our previous integrative analysis of two data sets would still generate consistent results. (Notice that the number of common genes was much reduced from 5216 to 2865 when the Stanford data set was included. This would change the number of selected pathways as shown above.) Figure 4 shows the scatter plot for the paired CES calculated based on two data sets and CES calculated based on three data sets (separately for up-regulated and down-regulated differential expression). For each plot, a clear correlation pattern can be observed. The Spearman correlation coefficients were both greater than 0.75 for these two plots (0.804 and 0.760). We also compared the listed of selected pathways with FDR < 0.05 (see above for details). For up-regulated differential expression, there were 92 pathways in common (among 224 selected based on two data sets and 99 selected based on three data sets); for down-regulated differential expression, there were 11 pathways in common (among 15 selected based on two data sets and 74 selected based on three data sets). If [(the number of commonly selected pathways)/(the number of smallest list of selected pathways)] was used as the overlap proportion, then we would have 92*/*99 = 92.9% and 11*/*15 = 73.3% as the overlap proportions for up-regulated and down-regulated differential expression, respectively. Therefore, a satisfactory consistency between both results was also observed.

**Figure 5:**
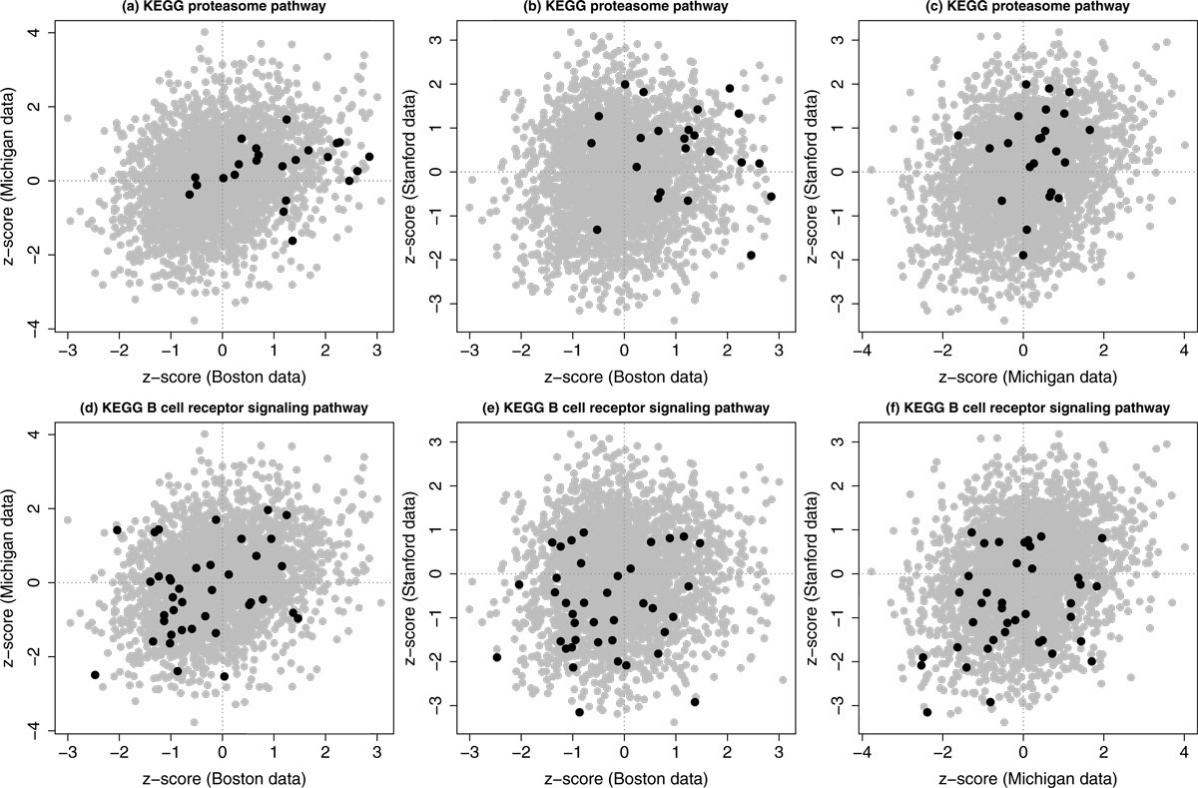
**Illustrative examples based on three data sets**. Two illustrative examples for our proposed method for a concordant integrative gene set enrichment analysis of three data sets. In each plot, the gray dots represent all paired z-scores for 2865 common human genes and the black dots represent the paired z-scores for the gene set specified in the title.

About two pathways mentioned particularly in our first application, there were two proteasome pathways in the Version 3.0 of the C2 canonical pathway collection: one given by BioCarta and the other given by KEGG. For both pathways, their CESs and FDRs for up-regulated differential expression were consistently respectively *>* 0.999 and *<* 0.001 based on our integrative analysis of two data sets, and these values were also consistently respectively *>* 0.95 and *<* 0.005 based on our integrative analysis of three data sets. There were also two BCR signaling pathways collected by KEGG and Signaling Gateway, their CESs and FDRs for down-regulated differential expression were consistently respectively *>* 0.95 and *<* 0.01 based on our integrative analysis of three data sets. Based on our integrative analysis of two data sets, the CES and FDR for the pathway by KEGG were respectively *>* 0.7 and *<* 0.2 and these two values for the pathway by Signaling Gateway were respectively *>* 0.9 and ~ 0.05. Figure 5 shows different paired *z*-scores from three data sets and the *z*-scores for these two pathways are highlighted for an illustration.

### KEGG cancer pathways

There is a collection of cancer pathways in the database of Kyoto Encyclopedia of Genes and Genomes (KEGG with web link http://www.genome.jp/kegg/). According to the database updated on July 24, 2013, 17 pathways are associated with lung cancer and general cancer studies. Table 2 lists 16 of these pathways that are also in the Version 3.0 of the C2 canonical pathway collection. (The KEGG PI3K-AKT signaling pathway is not included since it is not listed in the C2 collection. Notice that only pathways from KEGG are included. Pathways with same or similar names from other online databases like Reactome are not considered here. This ensures the consistency between the gene sets from the C2 collection and the gene sets mentioned in the KEGG cancer pathways.) Since a pathway could be enriched in either up-regulated or down-regulated differential expression, we would choose the one with larger CES if the absolute difference of two CESs was greater than 0.1 (same results observed when this threshold value was set between 0.05 to 0.15), which was a conservative choice of threshold value. Otherwise, we would not present any further analysis results for this pathway. For examples, if these two CESs were 0.5 (up-regulated) and 0.45 (down-regulated), then no further analysis results would be presented for this pathway; if these two CESs were 0.8 (up-regulated) and 0.1 (down-regulated), then the analysis results based on up-regulated differential expression would be presented. For these 16 pathways listed in Table 2, the results from the analysis described in our first and second applications were consistent. All the pathways except the TGF-*β* signaling pathway showed FDRs < 0.2 for at least one applications. Ten and eight pathways showed FDRs < 0.1 and FDRs < 0.05 respectively for at least one applications. Furthermore, all sixteen pathways showed FDRs < 0.25 for at least one applications.

**Table 2:** An exploration of KEGG cancer pathways.

		Two data sets			Three data sets		
**KEGG cancer pathways**	**U/D**	**CES**	**Diff.**	**FDR**	**CES**	**Diff.**	**FDR**
PPAR signaling *	down	0.671	*>*0.1	0.194	0.563	*>*0.1	0.210
MAPK signaling **	down	0.639	*>*0.1	0.209	0.857	*>*0.1	0.063
ERBB signaling *	up	0.629	*>*0.1	0.119	0.581	*>*0.1	0.188
Calcium signaling **	down	0.925	*>*0.1	0.051	0.694	*>*0.1	0.153
Cytokine-cytokine receptor interaction ***	down	0.717	*>*0.1	0.172	0.943	*>*0.1	0.022
Cell cycle ***	up	0.998	*>*0.1	*<*0.001	0.959	*>*0.1	0.012
p53 signaling ***	up	0.999	*>*0.1	*<*0.001	0.944	*>*0.1	0.018
MTOR signaling *	down	0.724	*>*0.1	0.167	0.611	*>*0.1	0.193
Apoptosis *	down		*≤* 0.1		0.776	*>*0.1	0.102
WNT signaling ***	down		*≤* 0.1		0.888	*>*0.1	0.048
TGF-*β* signaling	down		*≤* 0.1		0.521	*>*0.1	0.236
VEGF signaling ***	down	0.784	*>*0.1	0.136	0.919	*>*0.1	0.033
Focal adhesion ***	up	*>*0.999	*>*0.1	*<*0.001	0.830	*>*0.1	0.077
ECM receptor interaction ***	up	0.996	*>*0.1	*<*0.001	0.977	*>*0.1	0.005
Adherens junction *	up	0.646	*>*0.1	0.114		*≤* 0.1	
JAK-STAT signaling ***	down	0.875	*>*0.1	0.082	0.901	*>*0.1	0.044

Our application results for sixteen KEGG cancer pathways. "Diff" column presents the absolute difference between the CES based on up-regulated differential expression and the CES based on down-regulated differential expression. If "Diff"*≤* 0.1, then no further analysis results is presented. Otherwise, the larger CES as well as the related FDR and differential expression direction (up or down) are presented in the "CES", "FDR" and "U/D" columns, respectively. Both application results (an integrative analysis of two data sets and an integrative analysis of three data sets) are presented for the listed pathways. Pathways with symbols *, ** or *** means that FDRs < 0.2, FDRs < 0.1 or FDRs < 0.05 are observed for at least one applications, respectively.

## Conclusions

In this study, we proposed a mixture model based statistical method for the concordant integrative gene set enrichment analysis. Our method was first applied to two published lung cancer microarray gene expression data sets. As shown in Figure 1, gene sets like the proteasome and BCR signaling pathways were identified by our method. These gene sets were not identified in the previous study [8] since the differential gene expression among these gene sets were relatively weak. However, the concordant enrichment of these gene sets was detected by our method. This comparison illustrated the advantage of our proposed concordant integrative gene set enrichment analysis. The analysis results from our second application (a concordant integrative analysis of three data sets) also showed that many gene sets could be identified with low false discovery rates. A consistency between both results was also observed. A further exploration based on the KEGG cancer pathway collection demonstrated the practical usefulness of our proposed method. Overall, this study illustrates that we can improve detection power and discovery consistency through a concordant integrative analysis of multiple large-scale two-sample gene expression data sets.

There are several advantages for our proposed method. The genome-wide concordance can be statistically tested before the integrative analysis. The mixture model is estimated based on the maximum likelihood estimation procedure. Furthermore, our integrative analysis of gene sets is based on a probabilistic framework, which can be conveniently used for the calculation of false discovery rates. However, there are also limitations. Our proposed mixture model is simple and it contains only three components. Normal distributions are assumed for these components. Furthermore, we assume that different genes behave independently (Gold et al. [32] have showed that the independence assumption can be acceptable in practice). These limitations should be considered when our method is used in practice.

For our future research, it will be useful to extend our proposed method for an integrative analysis of data with multiple sample groups. This will be particularly useful for studying diseases with different progression stages. Although a major proportion of gene expression data have been collected for binary outcomes (e.g. normal vs. abnormal), data with other types of responses (e.g. survival data) have also been collected. It will also be useful to extend our method for these data. Furthermore, when our proposed method is used for an integrative analysis of more than 3 data sets, it is desirable to simplify the mixture model so that the number of model parameters (particularly for ) can be reduced to achieve statistical efficiency. Furthermore, we would also like to consider more robust approaches (e.g. a nonparametric method) to the concordant integrative gene set enrichment analysis.
